# Household Contact Screening and Yield of Tuberculosis Cases—A Clinic Based Study in Chennai, South India

**DOI:** 10.1371/journal.pone.0162090

**Published:** 2016-09-01

**Authors:** Dina Nair, Nandita Rajshekhar, Joel Shyam Klinton, Basilea Watson, Banurekha Velayutham, Jaya Prasad Tripathy, Mohideen Shaheed Jawahar, Soumya Swaminathan

**Affiliations:** 1 National Institute for Research in Tuberculosis, Chennai, India; 2 Mailman School of Public Health, Columbia University, New York City, United States of America; 3 International Union against Tuberculosis and Lung Disease, The Union South-East Asia Office, New Delhi, India; 4 Indian Council of Medical Research, New Delhi, India; Central University of Tamil Nadu, INDIA

## Abstract

**Background:**

Contact investigation is an active case finding strategy to increase detection of Tuberculosis (TB) and a key component of TB control programs. The household contacts are at a higher risk of exposure than members of the general population. The information on the value and yield of household contact screening and the approaches used in high incidence settings like India is limited.

**Objective:**

To evaluate the yield of active case finding in household contacts of newly diagnosed smear positive TB patients and the factors associated with increased yield.

**Method:**

Retrospective record review of the household contacts of newly diagnosed sputum smear positive patients (index case) enrolled in a clinical trial at National Institute of Research in Tuberculosis, Chennai during the period 2007–2014. A sequential screening algorithm with chest x-ray followed by symptom screen was employed to identify presumptive TB patients.

**Results:**

643 household contacts of 280 index TB patients were identified out of which 544 (85%) consented for screening. 71/544 (13%) patients had an abnormal chest radiograph and out of them 70% were symptomatic. A total of 29/544 (5.3%) contacts were found to have TB among whom 23/29 (79%) were sputum smear positive. The number needed to screen (NNS) to identify a new TB case among all household contacts was 19 and among those with an abnormal CXR was 02. Age group > 44 years, male gender and siblings of the index case was associated with abnormal chest radiograph whereas age group between 15–44 was significantly associated with developing TB disease among household contacts.

**Conclusion:**

Active screening among household contacts is an effective way to improve TB case detection. The yield for new TB cases among contacts with abnormal x-ray was high in this study and the use of Chest X-rays in combination with symptom screen is recommended.

## Introduction

Contact screening is a key component in national tuberculosis (TB) programs and is an active case finding strategy to identify more TB cases. It involves systematic screening of the contacts of known TB patients. It ensures early detection of disease and prompt initiation of treatment thus reducing the disease burden, the risk of TB transmission and poor treatment outcomes [1].

There is an increased risk of exposure to the disease causing organism among the household contacts of TB patients than the general population [2]. The risk of developing TB infection and disease in contacts relates to the infectiousness of the patient, duration of exposure and proximity [3, 4] and susceptibility of the contact [5, 6].

A systematic review has shown that among household contacts or other close contact of an index TB case, around 3.5–5.5% are found to have previously undiagnosed and active TB. Despite this potential benefit, routine contact investigation is performed rarely and inconsistently in resource-limited settings probably due to constraints in finance and human resources [7, 8].

Contact screening can be either passive or active. The various modalities used for the contact screening include symptom screening, chest radiography (CXR), sputum smear and culture examination, rapid molecular diagnostic tests–GeneXpert, tuberculin skin test (TST) and interferon-γ release assay (IGRA). Active screening may be done with either symptom evaluation or CXR, or with symptom evaluation and CXR combined in parallel or sequentially.

Recent data revealed that compared to screening by symptoms, CXR generally shows greater accuracy and less heterogeneity, but is resource intensive. The pooled sensitivity of CXR reading (98%) was higher than that of the symptom screens (87%). A sequential screening algorithm with chest X-ray as a second screen for TB symptomatic shows a sensitivity and specificity of 90% and 56% respectively [9, 10]. However, there is very limited information on the value and yield of contact investigations and the approaches used in high incidence settings like India.

The objective of our study was thus to evaluate the yield of active case finding in household contacts of newly diagnosed smear and culture positive tuberculosis patients and the factors associated with increased yield.

## Methods

### Study settings and design

The National Institute for Research in TB (NIRT), Chennai, is an internationally recognized institution in TB research in India. This was a retrospective record review of the household contacts (HC) of newly diagnosed sputum smear and culture positive pulmonary TB patients (index case).These index cases were enrolled in a clinical trial at NIRT during the period 2007–2014. The household contacts (HC) of these patients were screened to rule out TB disease as per the national guidelines [11].

For this study, an index case was defined as newly diagnosed sputum smear and culture positive adult HIV seronegative pulmonary TB patients. A household contact (HC) was defined as a person living with and sharing food from the same kitchen as the index case for a minimum of three months prior to diagnosis of TB disease of the index case. The yield of contact investigations was defined as the number of household contacts who were identified as new TB cases as a result of active screening of the household contacts.

The basic demographic details of the index case such as age, sex, body weight, duration of symptoms, family history of TB, smoking habit, sputum smear and culture grading and chest radiograph (CXR) were recorded. A list of household contacts was obtained from the index case during the initial house visit by the health staff. At the time of enrollment of the index case to the trial all the household contacts were advised to attend for screening and investigations at NIRT. Household contacts who were willing to be screened were registered and their demographic details such as age, sex, relationship to the index case, bodyweight were noted. The contacts were screened using a chest radiograph (CXR) and evaluated for any symptoms suggestive of TB. A postero-anterior view was done in all and an additional lateral view was done in children less than 14 yrs. The CXR was read by a panel of three doctors and a consensus decision was taken. An abnormal CXR with symptoms suggestive of TB were referred for sputum microbiological examination including smear, culture and drug susceptibility test (DST).The culture testing was done using the solid medium (Lowenstein Jenson) and DST was done by minimal inhibitory concentration (MIC) for Isoniazid, Rifampicin and Ethambutol and by Resistance Ratio (RR) method for Streptomycin [11]. In the absence of symptoms with an abnormal CXR a course of antibiotics was given and a repeat CXR was performed two weeks later to rule out persisting abnormality.

All the asymptomatic contacts were advised to attend the clinic if symptomatic at any time. Contacts with a normal CXR, but with persisting symptoms were evaluated for extra-pulmonary TB. The contacts who were diagnosed with TB disease were initiated on treatment as per the national guidelines.

All pediatric contacts less than 6 yrs of age were given INH prophylactic therapy (10mg/kg body weight) for a period of 6 months along with pyridoxine, after excluding active TB disease (standard of care in the national programme). They were followed up every month until 6 months and thereafter once in 3 months for 12 months.

This was a retrospective record review of the household contacts screened as per the national guidelines and standard of care. Specific ethical approval and individual written consent was therefore not obtained. The clinical trial was approved by a Scientific Advisory Committee and independent ethics committee and registered with the Clinical Trials Registry of India (CTRI/2008/091/000024).

### Statistical analysis

Data were entered and checked for errors in Microsoft Excel 2013. Statistical analysis was done using STATA/SE 9.1. Categorical variables were described in terms of frequencies and percentages. Chi-square test was used to examine associations between socio-demographic characteristics, and other covariates of interest with x-ray abnormality and TB disease among contacts. Univariate and multiple logistic regression analysis were used to determine the predictors of x-ray abnormality and TB disease among contacts. Variables entered into the multiple regression models were selected on the basis of significance in the univariate analysis. A 95% confidence interval and a 5% level of significance were used to interpret statistical significance. The p-values were adjusted for clustering at the household level. All statistical tests were two-tailed.

## Results

A total of 280 index TB cases were recruited. The demographic profile of the index cases is shown in **Table 1**. The index cases were predominantly males (70%) and most of the patients (77%) belonged to the age group 15–44. The mean body weight was 43 ±9 kgs. CXR showed cavitary lesion in 91 (33%) and 263 (94%) had a higher sputum culture grading (> = 2+). A total of 106 (38%) had history of smoking and 89 (32%) had history of TB in the family. All were symptomatic and 210 (70%) had cough for more than 4 weeks.

**Table 1:** Socio-demographic and clinical characteristics of new sputum smear positive index cases (N = 280) in Chennai, India.

Characteristic	No. of Patients	%
**Age (years)**		
< 45	216	77.1
> = 45	64	22.9
**Sex**		
Male	196	70.0
Female	84	30.0
Body Weight (Kg.)*		
< 45	172	61.4
> = 45	104	37.1
**X Ray Cavity**		
No	189	67.5
Yes	91	32.5
**Sputum Grading- Culture**		
< 2	17	6.1
> = 2	263	93.9
**Sputum Grading- Smear**		
< 2	49	17.5
> = 2	231	82.5
**Smoking**		
Never	174	62.1
Current & Past	106	37.9
**History of TB in family**		
No	191	68.2
Yes	89	31.8
**Cough (in weeks)**		
< 4	70	25.0
4–8	134	47.8
> 8	76	27.2

*Details missing in 4 patients.

A total of 643 contacts (an average of 2–3 contacts per index case) were registered during the house visits. The basic demographic details are given in **Table 2**. A total of 311(48%) were in the age group 15–44 and 111 (17%) were children less than 6 yrs of age. Around 59% of them were female contacts.

**Table 2:** Socio-demographic and clinical characteristics of house hold contacts of (N = 643) new sputum smear positive index cases in Chennai, India.

Criteria	No. of Contacts	%
**Age (years)**		
< 6	111	17.3
6–14	122	18.9
15–44	311	48.4
>44	99	15.4
**Sex**		
Male	264	41.1
Female	379	58.9
Relationship to the index case*		
Spouse	175	27.6
Parent	84	13.2
Child	281	44.2
Sibling	52	8.3
Grandchild	10	1.6
Other adult(>14)	25	3.9
Other child (< = 14)	8	1.2
**Contacts screened**	544	84.6
Abnormal CXR^a^	71	13.1
Past history of treatment^a^	17	3.1
Initiated on ATT^a^	29	5.3
Smear positives^	23	4.2
Smear negatives^	3	0.5
Extra-pulmonary TB^	3	0.5

*8 missing

^a^ % among those who were screened

^ % among those who were started on anti-TB treatment.

**Fig 1:**
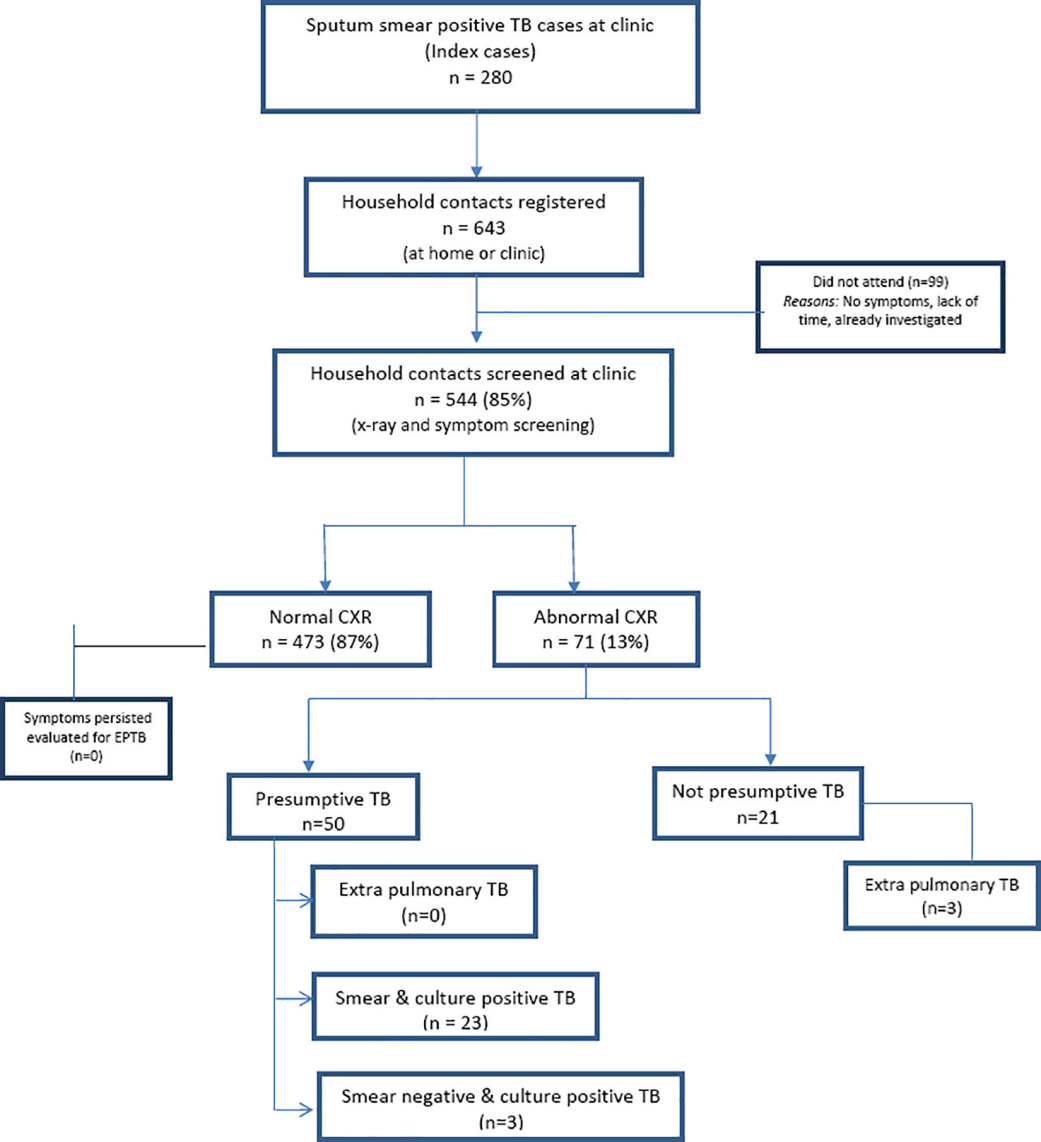
Flow chart of index TB cases and their household contacts, Chennai, India.

Among the contacts registered 544 (85%) presented themselves for CXR and symptom screening. Others did not attend due to various reasons (no symptoms, lack of time, already investigated, etc.) There were 71(13%) patients with abnormal CXR (consolidation, cavitary lesions, fibrosis, and pleural effusion). Among the contacts with abnormal CXR, 50 (70%) had symptoms suggestive of TB. Among the contacts screened 29 (5.3%) were diagnosed as new TB cases and initiated on anti-TB treatment (Category I). 23 (4.2%) contacts were new sputum smear positive pulmonary TB cases (**Fig 1**).

A multiple logistic regression model showed that age > 44 years (Adjusted O.R.: 5.5 [95% C.I.: 1.6, 19.0]), male gender (Adjusted O.R.: 2.2 [95% C.I.: 1.2, 3.9]) and siblings of the index case (Adjusted O.R.: 3.4 [95% C.I.: 1.2, 9.7]) were significant risk factors of abnormal CXR among household contacts.

Age group between 15–44 was a significant risk factor for developing TB disease while adjusting for other factors whereas sex and relationship to the index case were not found to be significant (**Table 3**).

**Table 3:** Factors associated with abnormal CXR and TB disease among the household contacts who had their CXR done (N = 544) in Chennai, India.

Characteristic	Total N	CXR Abnormal n (%)		Unadjusted OR (95% CI, p value)	Adjusted OR (95% CI, p value)	TB disease n (%)		Unadjusted OR (95% CI, p value)	Adjusted O.R. (95% CI, p value)
**Age in years**									
0–14	194	10	5	1.0 (Ref)	1.0 (Ref)	3	2	1.0 (Ref)	1.0 (Ref)
15–44	273	38	14	3.0(1.4–6.1, 0.003)	2.3(0.9–6.0, 0.09)	23	8	5.9 (1.7–19.8, 0.004)	7.3(1.4–36.7, 0.02)
>44	77	23	30	7.8(3.5–17.5, <0.001)	5.5(1.6–19.0, 0.01)	3	4	2.6(0.5–13.1, 0.25)	2.2(0.2–21.4, 0.5)
**Sex**									
Male	220	38	17	1.8(1.1–3.0, 0.02)	2.2(1.2–3.9, 0.01)	17	8	2.2(1.1–4.6, 0.04)	2.0(0.8–4.9, 0.14)
Female	324	33	10	1.0 (Ref)	1.0 (Ref)	12	4	1.0 (Ref)	1.0 (Ref)
Relationship to the index case*									
Spouse	155	17	11	2.3 (1.1–4.9, 0.04)	1.4 (0.5–3.9. 0.47)	5	3	1.5(0.4–5.4, 0.5)	0.7(0.2–3.0, 0.67)
Parent	64	18	28	7.3 (3.3–16.1, <0.001)	2.7 (0.8–9.2, 0.12)	4	6	3.1(0.8–11.8, 0.1)	3.2(0.5–19.9, 0.21)
Child	235	12	5	1.0 (Ref)	1.0 (Ref)	5	2	1.0 (Ref)	1.0 (Ref)
Sibling	47	11	23	5.7(2.3–13.8, 0.001)	3.4 (1.2–9.7, 0.02)	6	13	6.7(2.0–23.1, 0.002)	2.6(0.7–10.0, 0.16)
Others**	37	8	22	5.1(1.9–13.6, 0.001)	3.7 (1.3–10.8, 0.02)	4	11	5.6(1.4–21.8, 0.01)	4.1(1.0–17.2, 0.06)

* Details missing in 6 contacts

**Included other second degree relatives (Grandchild, cousin, uncle).

Association of various characteristics of the index case and yield of TB disease among contacts showed that past history of TB in the family was a significant associated factor (**Table 4**). Contacts of index cases who had a history of TB in the family had 2.5 (95% C.I.: 1.1, 5.7) times the risk for TB disease as compared to those without a family history of TB.

**Table 4:** Index case factors associated with TB disease among their house hold contacts (N = 544) in Chennai, India.

Characteristics Index cases	Contacts	TB disease		No TB disease		O.R (95% C.I.)	p-value
	N	n	%	n	%		
**CXR cavity**							
No	364	20	5.5	344	94.5	1.1 (0.5, 2.7)	0.82
Yes	180	9	5.0	171	95.0	1.0(Ref)	
**Sputum Smear**							
< 2	94	4	4.3	90	95.7	1.0(Ref)	
> = 2	450	25	5.6	425	94.4	1.3(0.4, 5.0)	0.68
**Sputum Culture**							
< 2	21	2	9.5	19	90.5	1.9(0.5, 7.9)	0.36
> = 2	523	27	5.2	496	94.8	1.0(Ref)	
**Smoking**							
Never	357	17	4.8	340	95.2	1.0(Ref)	
Current & Past	187	12	6.4	175	93.6	1.4(0.6, 3.1)	0.45
**H/o of TB in family**							
No	391	15	3.8	376	96.2	1.0(Ref)	
Yes	153	14	9.2	139	90.8	2.5(1.1, 5.7)	0.03
**Cough (avg. weeks)**							
< 4	125	11	8.8	114	91.2	2.1(0.8, 5.4)	0.11
4–8	278	12	4.3	266	95.7	1.0 (0.3, 2.8)	0.98
>8	141	6	4.3	135	95.7	1.0(Ref)	

The number needed to screen (NNS) to identify one new case of TB among all household contacts was 19. NNS was found to be 02 for household contacts with an abnormal CXR and contacts with both presumptive TB symptoms and an abnormal CXR. (**Table 5**).

**Table 5:** Number of household contacts of TB index cases needed to screen (NNS) to identify one new case of TB/smear positive TB, Chennai, India.

	Household contacts screened	New TB case	NNS for New TB case	New smear positive TB	NNS for new smear positive TB
**Total**	544	29	19	23	23
**Abnormal CXR**	71	29	02	23	03
**Abnormal CXR & presumptive TB**	50	26	02	23	02
**Children 0-14y**					
**Total**	194	03	65	02	97
**Abnormal CXR**	10	03	03	02	05
**Abnormal CXR & presumptive TB**	7	02	03	02	03
**Adults**					
**Total**	350	26	13	21	17
**Abnormal CXR**	61	26	02	21	03
**Abnormal CXR & presumptive TB**	43	24	02	21	02

TB = tuberculosis; NNS = number needed to screen.

Evaluation of x-ray as a screening tool for household contacts of Pulmonary TB patients was determined taking smear positivity as the gold standard. The sensitivity was 96% (95% C.I.: 79.7–99.9) and specificity was 90.9% (95% C.I.: 88.1–93.3).

Culture and drug susceptibility testing of the isolates were available for 12 smear positive contacts. The drug susceptibility pattern showed susceptibility to Isoniazid, Rifampicin, Ethambutol, and Streptomycin which was similar to that of the index cases.

## Discussion

This study reports the results of screening of household contacts of smear-positive HIV sero-negative pulmonary TB index cases. The overall yield for active tuberculosis cases was 5.3% which was low among children <15 years with 1.5%. However the yield for new TB cases among contacts with abnormal x-ray was high (41%). Many studies have shown that active case finding among household contacts yields substantially more TB cases than passive case detection [12,13,14,15]. The yield for active TB case finding through contact investigations ranged from 0 to 6.9% among household contacts in high burden countries [16, 17]. The overall yield for active screening was 5.3% in our study which is less when compared to the findings of a similar study conducted in Chhattisgarh, India with an yield of 9.0% [18].

Hoog et al. found higher sensitivity and accuracy with CXR screening compared to symptom screening alone. However, a combination of CXR and symptom screening offers the highest sensitivity for identification of presumptive TB patients [9]. The number of contacts with abnormal x-ray needed to screen to find a new case of TB was unusually low (02), indicating a very effective strategy. In another study at Malawi only 8% of smear-positive TB and 31% of either smear- or culture-positive disease had normal CXR. Conversely, 27% of patients who had smear- and culture-negative sputum had a CXR suggestive of TB [13]. In our study, though few in numbers, CXR was abnormal in almost all the household contacts who developed the disease. The utility of CXR as an alternative modality in contact screening investigations in terms of feasibility and cost effectiveness should be explored further. We also observed a higher proportion of abnormal CXR in adults above 45 years. This may be due to age-related changes, such as loss of supporting tissue resulting in enlargement of the distal airspaces [19]. Cost-effective analysis of various screening strategies needs to be done. In the light of the End TB Strategy a more aggressive approach is warranted if we are to end TB in our time [20].

The risk of infection among contacts depends on several factors such as the distance of the source case from the contact during exposure, the duration of exposure and other environmental factors [21]. First-degree relatives are up to five times more likely to cause infection in contacts [22]. However in the present study, the effect was not statistically significant among first degree relatives (parents and siblings). More research is warranted to identify the various sociological and environmental factors for transmission in a larger group of vulnerable population.

All the contacts who developed the disease were incident cases. History of TB in the family of the index case had a higher risk of TB disease in the contacts. There are several explanations for this association such as HLA association [23], variation in the natural resistance-associated macrophage protein 1 (NRAMP1) gene [24] or the Mendelian predisposition [25]. But the role of genetics in TB still remains a topic for further research. A more detailed understanding of the genetic susceptibility to TB is needed to support development of new vaccines and therapies.

The study had several strengths. First, CXR and symptom screen was available for all the contacts who developed the disease. Second, the fact that the study was a part of an ongoing clinical trial ensures robust methodology and systematic data collection. Third, the study findings were reported in accordance with the STROBE guidelines [26]. Our study had few limitations as well. The study was done in a clinic setting among the patients registered under a clinical trial, hence may not be a representative sample of the population. Also the focus was only on active TB disease and not on TB infection due to the difficulty of discerning between remote and recent TB infection. We could not ascertain the BCG immunization status of contacts and also the other co-morbid conditions like HIV, diabetes and malnutrition. No information was available on participants who did not consent to participate; it is likely that those participants were different from the consenting ones. For those contacts who developed TB, we did not perform genotyping of index and contact pairs to ascertain whether transmission occurred in the household or community.

To conclude, the yield for new TB cases among contacts with abnormal x-ray was very high with the number needed to screen being unusually low i.e. 02, indicating a very effective screening strategy. Thus, we recommend the use of x-rays alone or in combination with symptom screen for household contact screening to identify new TB cases.
